# Quantitative influence and performance analysis of virtual reality laparoscopic surgical training system

**DOI:** 10.1186/s12909-022-03150-y

**Published:** 2022-02-10

**Authors:** Peng Yu, Junjun Pan, Zhaoxue Wang, Yang Shen, Jialun Li, Aimin Hao, Haipeng Wang

**Affiliations:** 1 State Key Lab of VR Tech & Syst, Beihang University, Beijing, China; 2 Pengcheng Laboratory, Shenzhen, China; 3 Beijing Normal University, Beijing, China; 4 Beijing General Aerospace Hospital, Beijing, China

**Keywords:** VR, Laparoscopic surgical skills training, Cognitive load, Flow experience, Depth perception

## Abstract

**Background:**

Virtual reality (VR) surgery training has become a trend in clinical education. Many research papers validate the effectiveness of VR-based surgical simulators in training medical students. However, most existing articles employ subjective methods to study the residents’ surgical skills improvement. Few of them investigate how to improve the surgery skills on specific dimensions substantially.

**Methods:**

Our paper resorts to physiological approaches to objectively study the quantitative influence and performance analysis of VR laparoscopic surgical training system for medical students. Fifty-one participants were recruited from a pool of medical students. They conducted four pre and post experiments in the training box. They were trained on VR-based laparoscopic surgery simulators (VRLS) in the middle of pre and post experiments. Their operation and physiological data (heart rate and electroencephalogram) are recorded during the pre and post experiments. The physiological data is used to compute cognitive load and flow experience quantitatively. Senior surgeons graded their performance using newly designed hybrid standards for fundamental tasks and Global operative assessment of laparoscopic skills (GOALS) standards for colon resection tasks. Finally, the participants were required to fill the questionnaires about their cognitive load and flow experience.

**Results:**

After training on VRLS, the time of the experimental group to complete the same task could drop sharply (*p* < 0.01). The performance scores are enhanced significantly (*p* < 0.01). The performance and cognitive load computed from EEG are negatively correlated (*p* < 0.05).

**Conclusion:**

The results show that the VRLS could highly improve medical students' performance and enable the participants to obtain flow experience with a lower cognitive load. Participants' performance is negatively correlated with cognitive load through quantitative physiological analysis. This might provide a new way of assessing skill acquirement.

**Supplementary Information:**

The online version contains supplementary material available at 10.1186/s12909-022-03150-y.

## Background

As one of the modern minimally invasive procedures, laparoscopic surgery has become popular primarily due to its minor wounds and rapid recovery. However, residents generally have a long training period (6 years at least) to be qualified laparoscopic surgeons [1]. Traditional laparoscopic surgical training usually conducts experiments on animals or corpses organs, leading to adverse effects, such as high cost, low reusability, and related ethical issues [2]. The advent of virtual reality (VR) based laparoscopic surgery simulators (VRLS) has changed the surgeons learning mode. It not only reconstructs the natural surgical environment and procedures but also can be reused for a variety of designed training tasks without surgical risk [3]. Many researches validate the effectiveness of VRLS on training surgeons [4]. To our knowledge, most of them employ subjective methods to study the improvement of medical students’ surgical skills through VRLS. But few papers investigate how to substantially improve the surgery skills on specific dimensions, such as physiological and psychological perspectives.

In 1988, Sweller discussed the cognitive load theory (CLT) thoroughly and systematically for the first time based on resource-limited theory and schema theory [5]. They believe that cognitive load refers to the total amount of mental activities exerted on an individual’s cognitive system during a specific time of operation. CLT is a vital learning theory, which is paid more and more attention in medical education. The medical field is a complex knowledge field. Medical workers must simultaneously integrate various knowledge and skills at a specific time and make quick responses and decisions, which is prone to excessive cognitive load or even overload. Flow experience is the state of mind in which a person is fully engaged in some activity and reaches an extreme level of pleasure [6]. For example, in the case of laparoscopic surgery, flow is the physician's total mental commitment to the ongoing procedure and the creation of a consciousness state to perform with their best surgical abilities. They find that when people maximize their physical and mental conditions, they often produce the ultimate optimal experience [6] . Studies show that improving flow experience during laparoscopic surgery can enhance the effectiveness of the operation, thereby enhancing patient safety [7].

To our knowledge, there is no research to directly and quantitatively explore the relationship between the flow and total cognitive load. However, some studies have shown that learners with good academic performance have higher flow experience, lower external cognitive load, and higher related cognitive load [8]. Chang et al.’s research has shown that flow experience is related to three different cognitive loads. It confirms that media richness and game interaction can improve learners’ flow experience, reduce external cognitive load, and promote closely related cognitive load [9].

The main advantages of the psychophysiological measurement of cognitive load are objectivity, sensitivity to different cognitive processes, non-interference of the program, implicitness, and continuity [10]. Electroencephalogram (EEG) is considered a physiological indicator, which can be used as an online and continuous cognitive load measurement method to detect subtle fluctuations in instantaneous load. The results of Başar et al. [11] and Gevins et al. [12] provide the feasibility tests for using EEG-based methods for monitoring cognitive load. Many scholars develop various flow scales based on the characteristics and needs of VR learning situations. However, a sense of control, immersion, clear goals, and feedback are still indispensable dimensions in the measurement of flow experience [13, 14]. At present, there are few studies evaluating flow experience in VR based on physiological indicators. The most commonly used methods to measure flow experience are retrospective questionnaires and interviews [15, 16]. Flow experience can be expressed in physical and physiological characteristics as an objective flow indicator [16] . In terms of EEG, some studies indicate the correlation between EEG and flow experience under peak performance conditions, and the induction of flow experience can improve the performance of workplaces, sports fields. The most easily acquired physiological data is the heart rate. Besides, the cognitive load is measured using EEG. Three hypotheses are designed as follows:

- H1: Training on VRLS could improve the performance of medical students in some dimensions.
- H2: Training on VRLS could improve the flow experience and lower the cognitive load for medical students.
- H3: The performance is positively related to flow experience and negatively associated with the cognitive load.

These hypotheses are validated through the following experiments and user studies. This work provides two main contributions:

1. Using multimodal sensing data (EEG and heart rate), a physiological approach is designed to measure the influence of VRLS on medical students;

(2) The experiments reveal the negative correlation between the skill performance of trainees and their cognitive load. After correlation analysis. This research can identify the potential benefits of VRLS in improving the surgical skills of medical students during laparoscopic procedure training.

## Methods

### Participants

Fifty-one participants between 17 and 27 years old (22.30±2.79) were recruited in this study. The hospital ethics committee approved our experiments. There were 20 male and 31 female medical students. 2 persons were left-handed, and the others were right-handed. 41 participants never experienced VR, and 10 participants have played VR or augmented reality (AR) games once or twice before this study. 15 persons (29.41%) played games on PC or mobile phones every day. Just 8 (15.69%) participants rarely played games in their daily life. All participants had never experienced a VR-based laparoscopic surgery simulator before this study. Besides, all participants did not operate on any laparoscopic surgery before. All participants were divided into two groups. The first group (control group) consists of 10 participants (5 male and five female). The second group (experimental group) consists of 41 participants (15 male and 26 female).

### Platform

**Fig. 1:**
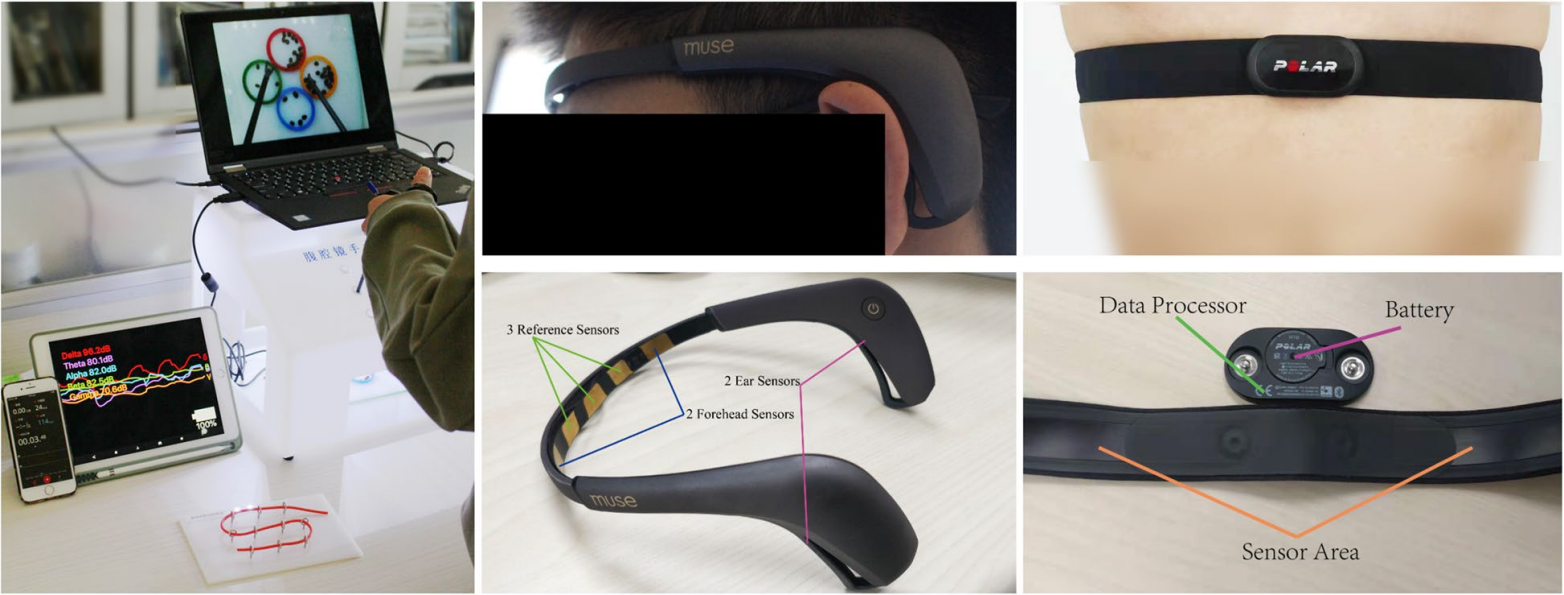
(Left) The participants are training on a box. The training process is recorded and saved as videos. The EEG is recorded using an iPad and the heart rate is recorded using an iPhone. (Middle upper) A participant wore a Muse 2 headband to measure his EEG when operating the surgery skill task. (Right upper) A participant wore a Polar H10 band to measure his heart rate. (Middle lower and Right lower) The configuration of Muse 2 and Polar H10

**Fig. 2:**
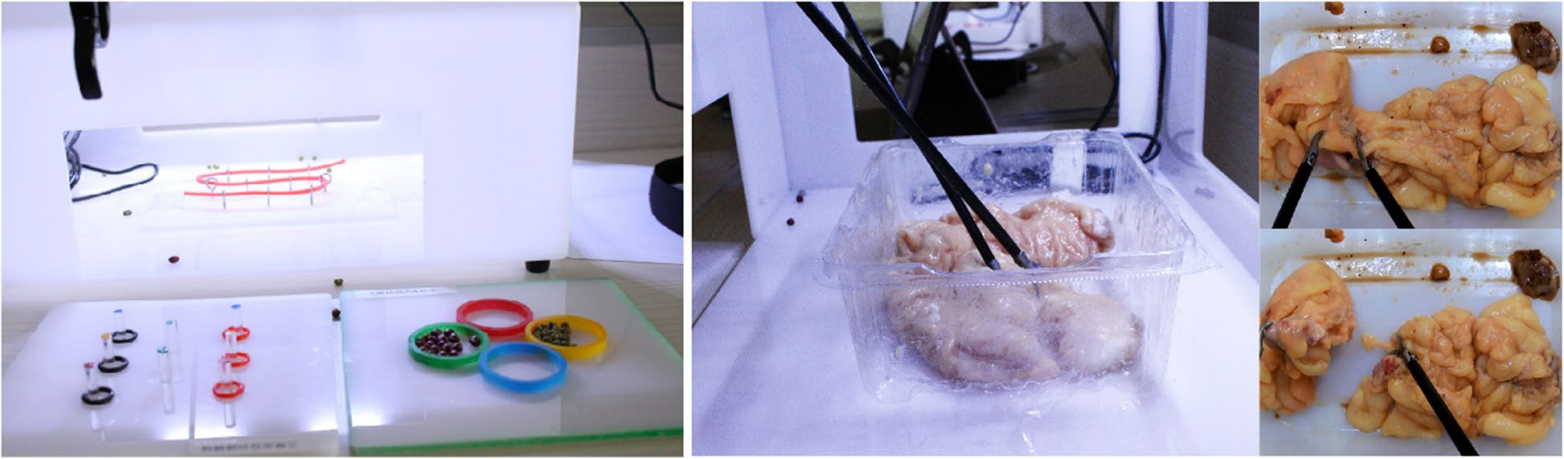
Three fundamental surgery tasks: peg transfer, picking beans, a threading skill practice (Left), and the colon resection task (Right)

The pre-test and post-test were carried out using a training box. The commercial laparoscopic physical training box (38×27×27 cm) is illustrated in Fig. 1. A camera was placed in the training box. As illustrated in Fig. 2, the participants were required to conduct four tests (three fundamental laparoscopic surgery skill training tasks: peg transfer, picking beans, and threading skill practice, one colon resection task). As shown in Fig. 1, the electroencephalography (EEG) data were collected using a four-channel dry electrode headset (Muse 2, InteraXon Inc.). The heart rate was recorded using a Polar H10 heart rate monitor chest strap.

**Fig. 3:**
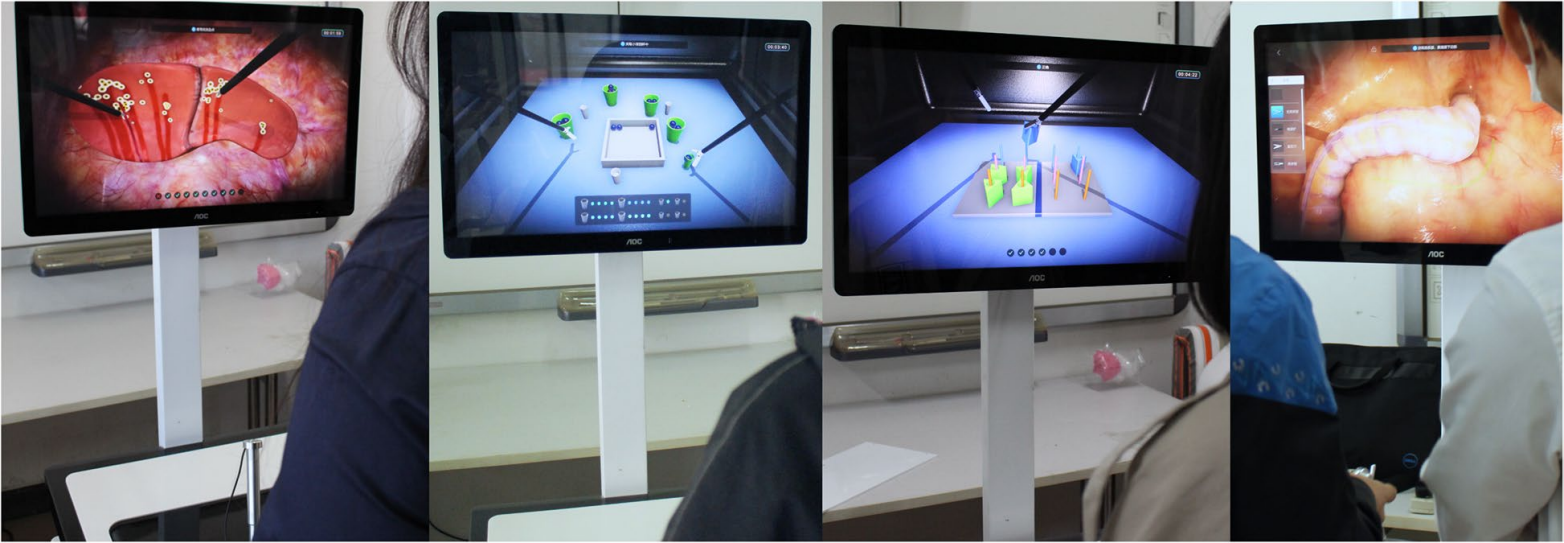
Training on VR laparoscopic surgery simulator. There are four training tasks on VLRS (from upper left to lower right): fixed point hemostasis, peg transfer, picking beams and colon resection

The VR laparoscopic simulator (Fig. 3) was developed by the State Key Lab of VR Tech & Syst at Beihang University. The simulator consists of two major components. The first component is the computation module, a high-performance PC connected with a touch-screen monitor. The second component is the simulation module, which contains two surgical handlers connected with haptic devices and a navigation camera in a box. Two-foot pedals can be utilized to activate electrosurgical coagulation during surgery training.

### Study design

The whole experiment consists of three main steps. Firstly, all participants were required to conduct a pre-test on a training box. The pre-test contains three fundamental surgery skills tasks (Pre-Fundamental Task, Pre-FT) and one colon resection task (Pre-Colon Resection Task, Pre-CRT). The EEG, heart rate, and the whole procedure were recorded. Secondly, the experimental group was asked to conduct the same kind of tasks on VRLS. Everyone had to complete four trials within a week, and each test lasted about 30 minutes. The control group did not do any surgery-related training. Finally, all participants conducted the post-test (Post-FT and Post-CRT) which is the same as the pre-test.

After finishing all experiments, participants were asked to complete four questionnaires regarding the cognitive load and flow experience. To explore the influence of VRLS on participants, three classical scales (Pass [14] NASA-TLX [17], WP Scale [18]) were used to measure the cognitive load. To calculate the flow experience during the experiments, the EGame scale [13], Cheng’s scale [19] were combined and redesigned questions according to our investigations. We analyzed the validity of the questionnaire and then conducted the pretest, reliability test, and validity test. In this questionnaire, Cronbach’s alpha coefficient reaches 0.804 (baseline: 0.6), while the KMO and Bartlett coefficients are 0.729 and sig=0.000. The scale’s reliability is satisfactory, ensuring the solidness and fitness of instruments.

### Data processing

#### Data collection

In this study, three types of data were obtained. The first was the performance scores computed from recorded videos according to the Global operative assessment of laparoscopic skills (GOALS) standards for colon resection tasks and our designed measure rules (e.g., completion time, number of mistakes, etc.) for fundamental surgery skill tasks. Five medical experts were invited to evaluate the performance scores anonymously by watching all participants’ videos. The final performance score of each participant was an average of 5 performance scores. The second was the self-reported scores, including cognitive load scores and flow experience scores computed from questionnaires. The third was the physiological data extracted from heart rate data and EEG. In educational psychology, EEG can measure the neuronal response of changing levels of cognitive stimuli, making it the most suitable measure for cognitive load assessment. EEG has been used for measuring the cognitive load of tasks and data analysis for over a decade [1].

The performance scores and physiological data need to be processed before getting meaningful information. The fundamental surgery skill tasks and colon resection tasks were measured from different dimensions for performance scores. The performance of fundamental surgery skills was measured from 7 dimensions, including the completion time, the number of failures in peg transfer and picking beans, the number of times rope dropped, motion smoothness, depth perception, and bimanual dexterity. The GOALS standard measured one’s laparoscopic skills from 4 aspects: depth perception, bimanual dexterity, efficiency, tissue handling, and autonomy.

After filtering EEG data according to four data quality indicators (1, Good, 2, Medium, >=3, Bad), the physiological data could be processed from the time, frequency, and nonlinear domains. We computed each participant’s average, minimum, maximum heart rate for heart rate data. To study the cognitive load change between pre and post-test, the participants’ cognitive load score was computed using EEG data according to [18]. Here's the process of extracting cognitive load from EEG, segmented into Baseline and Stimulus Epochs. These epochs were then processed using the S-Transform for each sensor. The resulting time-frequency planes were further processed to extract the gravity frequency and energy density for the theta and alpha bands of frequencies in each epoch. These values were combined in the Cognitive Analysis, resulting in a single time series of cognitive load for each sensor. These time series were then combined through spatially aware averaging to form the overall cognitive load for the trial

#### Statistical analysis

After pre-processing all data, SPSS (Statistics V.25) was utilized to analyze our computed scores. We used the classical paired samples t-test to test the differences between pre and post-tasks. A *p*-value of < 0.05 was recognized as statistically significant.

## Results

In this section, the influence of VRLS on participants was investigated from two aspects: performance (Sec. 3.1) and physical-psychological (Sec. 3.2, Sec. 3.3). Besides, their correlations were revealed in Sec. 3.4.

### Performance and VRLS

**Fig. 4:**
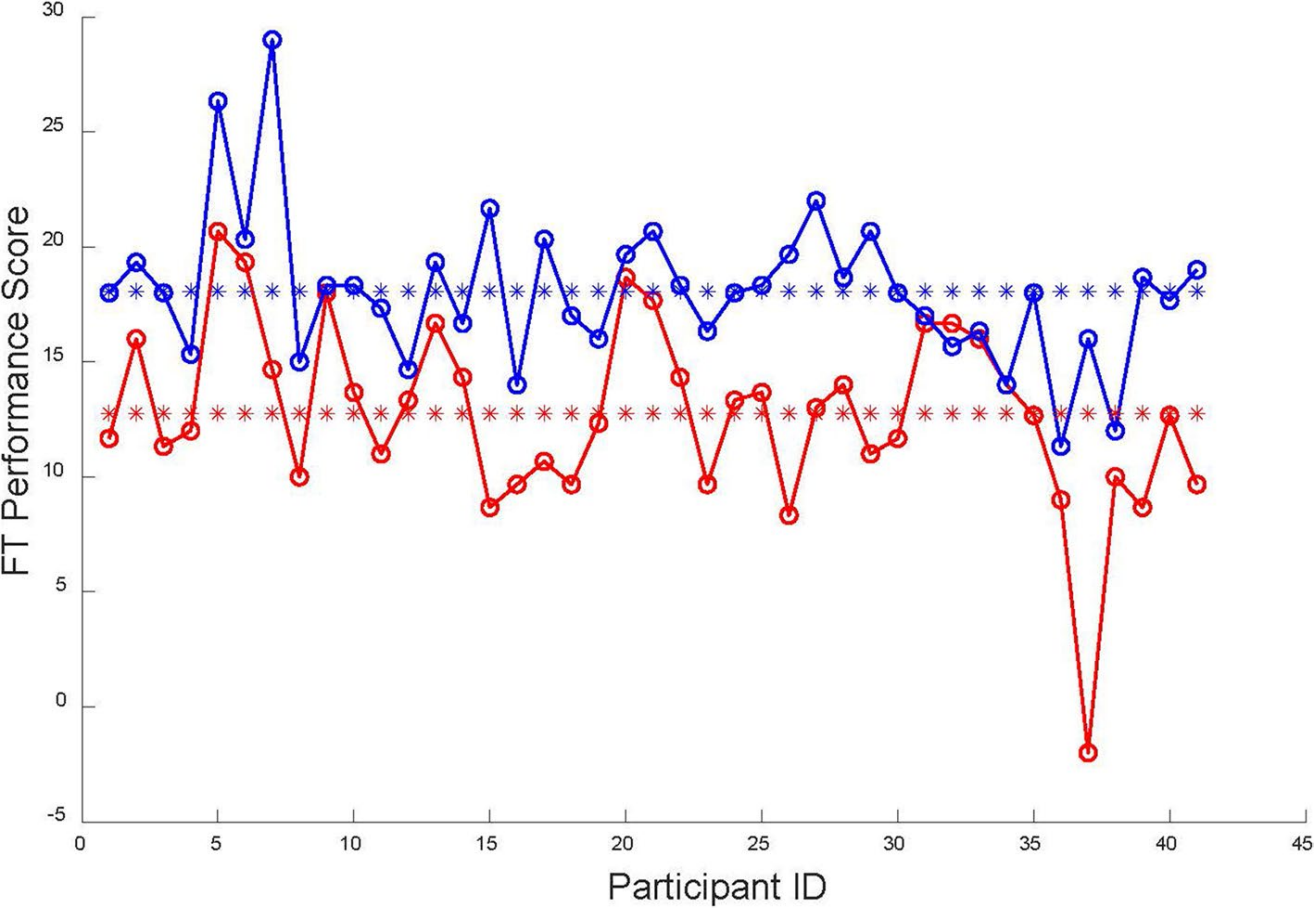
Pre-FT and Post-FT performance comparison. Pre-FT performance scores of each participant (red circles lined by red lines), Pre-FT average performance scores 12.76 ± 3.96 (red stars). Post-FT performance scores of each participant (blue circles lined by red lines), Post-FT average performance scores 18.07 ± 3.24 (blue stars). The significance of the difference between Pre-FT and Post-FT is *p* ≤ 0.01

**Fig. 5:**
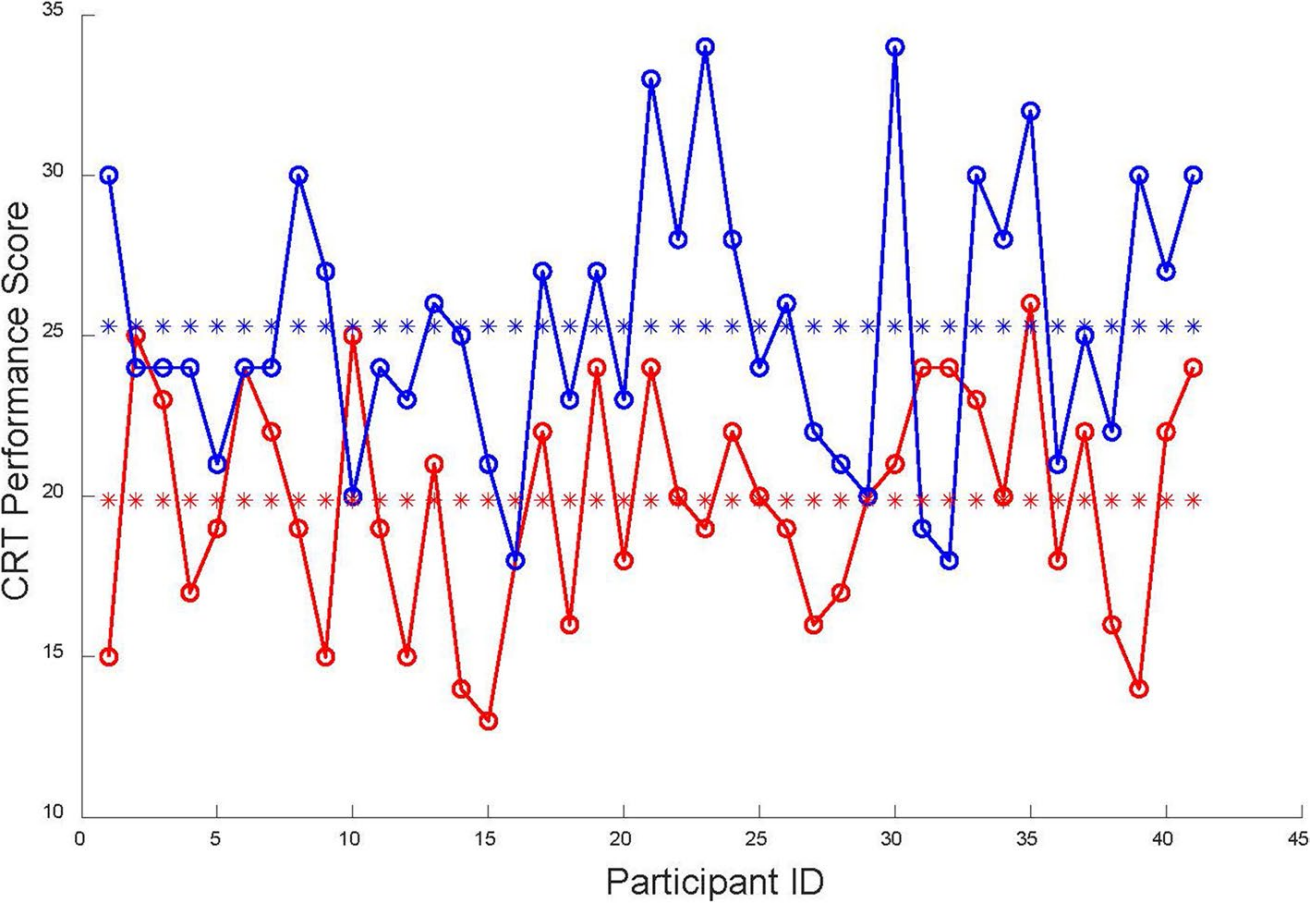
Pre-CRT and Post-CRT performance comparison. Pre-CRT performance scores of each participant (red circles lined by red lines), Pre-CRT average performance scores 19.88 ± 12.51 (red stars). Post-CRT performance scores of each participant (blue circles lined by red lines), Post-FT average performance scores 25.30 ± 18.16 (blue stars). The significance of the difference between Pre-CRT and Post-CRT is *p* < 0.01

After training on VRLS, the time of the experimental group to complete the same task could drop sharply *p* < 0.01. The first column of Table 1 shows the time required for each participant to complete each task. The efficiency of participants improved 1.6 and 5.4 times for the fundamental surgery task and colon resection task, respectively. The time to complete the same task did not drop significantly (*p* > 0.1) in the control group. Besides, the performance scores for the fundamental surgery skill task (FT) and colon resection task (CRT) are shown in Fig. 4 and Fig. 5. The Pre-FT score was significantly lower than the Post-FT score (*p* < 0.01). We obtained the same results for the colon resection task. In the colon resection task, the participants’ performance was enhanced in all four dimensions of GOALS standards (*p* < 0.001).

**Table 1:** The experimental group’s statistical heart rates (HR) results

	Avg Time(min)	Avg HR	Min HR	Max HR
Pre-FT	21.95	94.96 (±11.14)	79.40(±10.24)	112.33(±10.65)
Pre-CRT	4.87	96.51(±11.48)	85.49(±10.39)	107.30(±12.05)
Post-FT	14.07	92.71(±11.84)	81.30(±10.35)	108.10(±12.52)
Post-CRT	2.59	92.67(±11.67)	85.10(±10.43)	101.26(±12.70)

### Flow experience

The flow experience is related to moderate heart rate (HR) [20]. Thus, the flow experience was investigated through self-reported psychological flow questionnaires and heart rates. The average heart rate decreased significantly (*p* < 0.05). Especially, the maximum heart rate dropped at the post-test procedure (*p* < 0.05).

The results of the self-reported flow experience are shown in Table 2. It demonstrated that the goals of tasks are clear (4.02/5), the participants had a positive attitude toward the whole experiment, and they had a great sense of involvement (4.19/5). From the aspect of flow dimension, the score is 3.95/5.0±0.96, which is a relatively high score.

**Table 2:** Descriptive statistics of the scores on flow experience after the experiments

	Mean	SD
VR Interactivity	3.85	0.89
Challenge	3.55	1.25
Skill	3.68	0.92
Goal Clarity	4.02	0.78
Knowledge Improvement	3.72	1.03
Positive Affect	4.29	0.77
Vividness	3.15	1.13
Involvement	4.19	0.75
Flow	3.95	0.96

Note: The questionnaire for measuring flow was constructed as a 5-point Likert scale (1 = “not at all true” to 5 “totally true”). Each row in the table represents the mean and standard deviation of a particular dimension of the flow experience of all participants in the experimental group

### Cognitive load

**Fig. 6:**
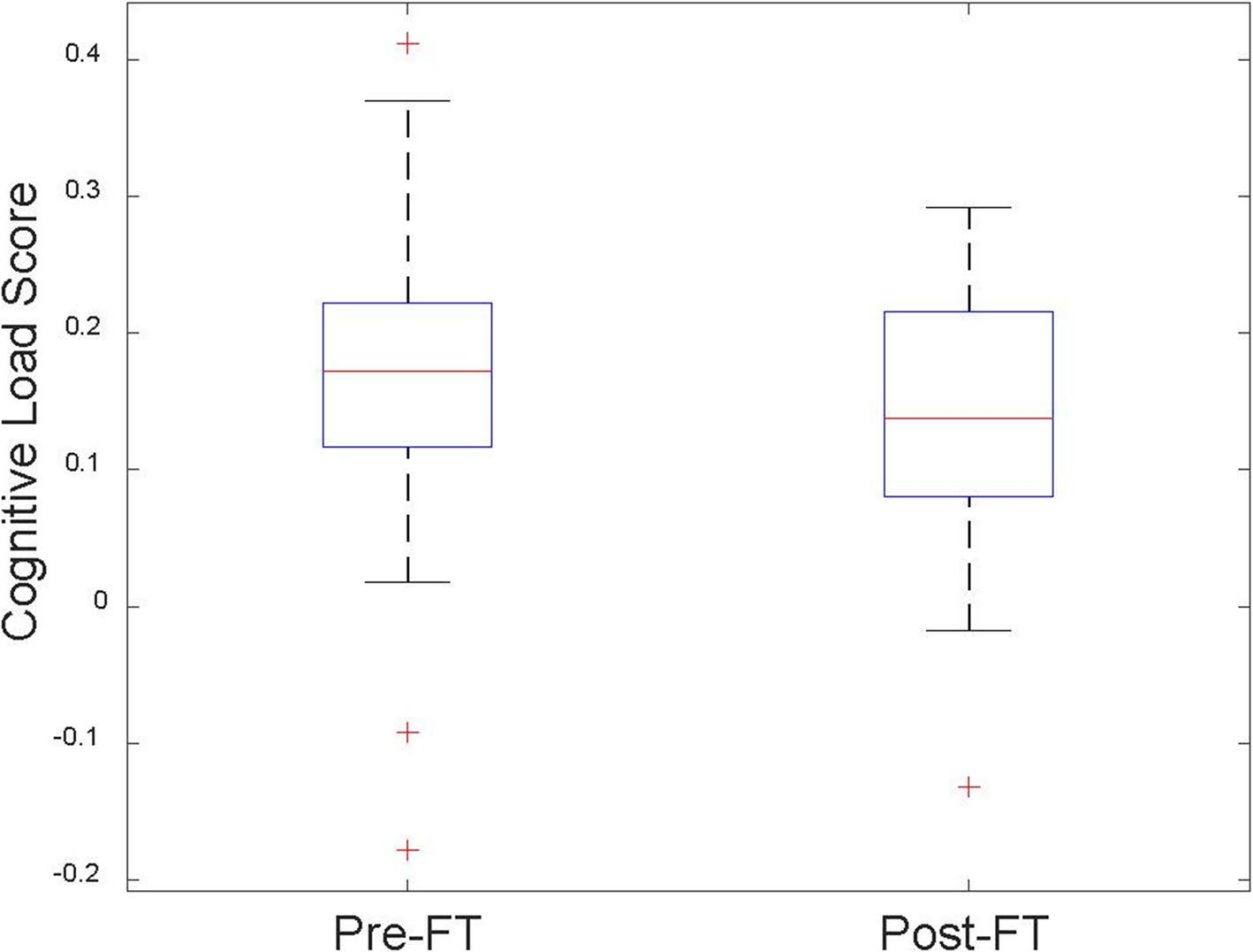
Pre-FT and Post-FT cognitive load score comparison. Pre-FT average cognitive load scores 0.17 ± 0.11, Post-FT average cognitive load scores 0.14 ± 0.09. The significance of the difference between Pre-FT and Post-FT using t-test is *p* = 0.04 < 0.05

**Fig. 7:**
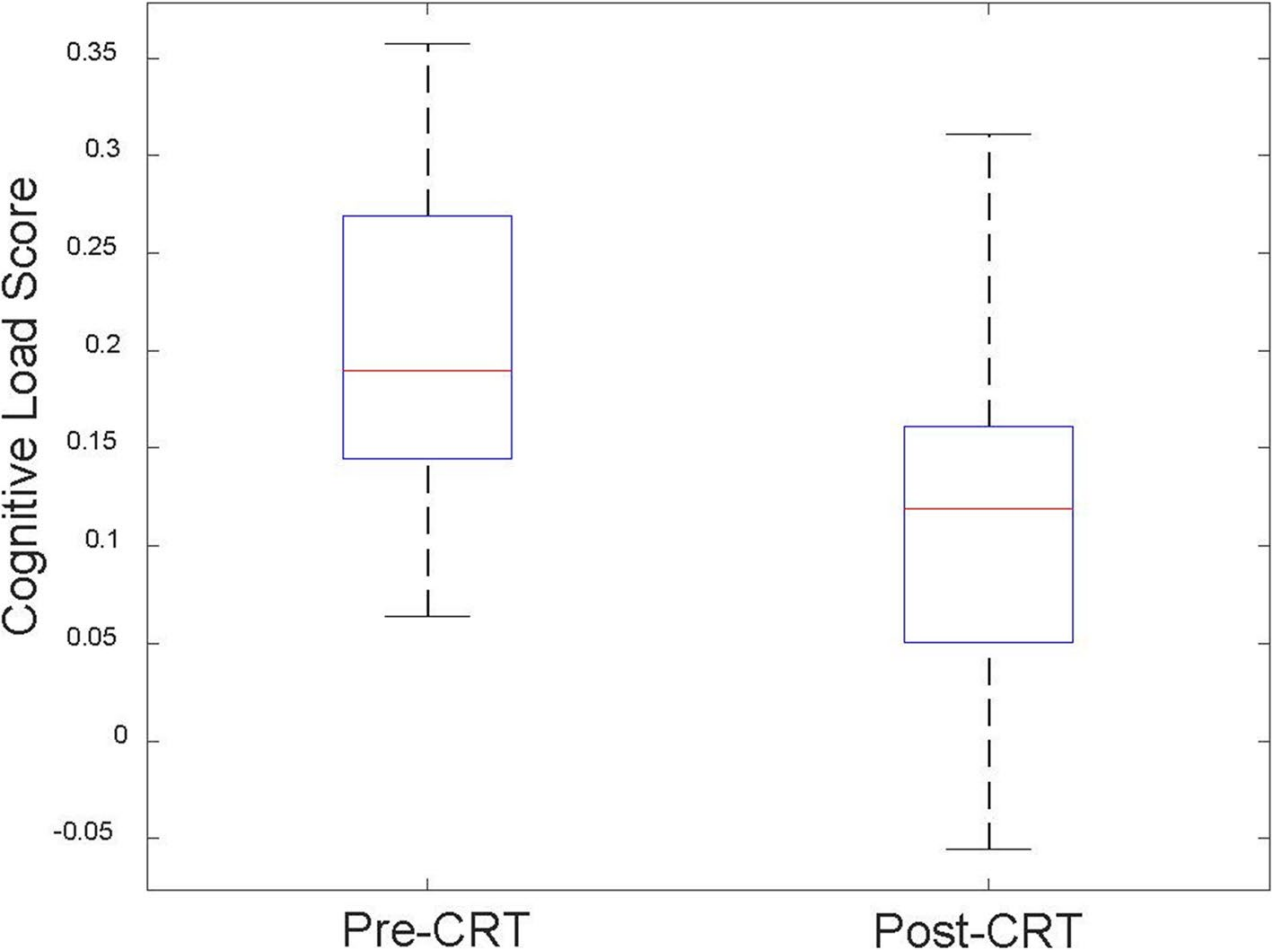
Pre-CRT and Post-CRT cognitive load score comparison. Pre-CRT average cognitive load scores 0.19 ± 0.01, Post-CRT average cognitive load scores 0.11 ± 0.01. The significance of the difference between Pre-CRT and Post-CRT using t-test is *p* = 0.0004 < 0.01

The overall cognitive was measured using Pass scale [14] (M = 6.09/10, SD = 1.32). The overall task difficulty was relatively low (M = 3.80/10, SD = 1.44). Table 3 (NASA-TLX) shows the self-reported mental workload of our designed tasks. The overall cognitive load of the experimental group was lower than the midpoint of the full range (0−10). Table 3 (WP Scale) demonstrated that the most important ability needed in laparoscopic surgery skills was depth perception (M = 8.12, SD = 1.82). The audio processing ability was the least used perception capability (M = 4.32, SD = 2.56). Fig. 6 shows the cognitive load scores comparison between pre (M = 0.17, SD = 0.11) and post (M = 0.14, SD = 0.09) fundamental surgery skills tasks. The post cognitive load was significantly lower than the cognitive load of Pre-FT (*p* < 0.05). Fig. 7 shows that the cognitive load scores were also significantly decreased in colon resection tasks (*p* < 0.01).

**Table 3:** Descriptive statistics of cognitive load after the experiment

	Measure Dimension	Mean	SD
NASA-TLX	Mental Demand	5.00	1.67
	Physical Demand	5.34	2.44
	Temporal Demand	4.07	2.16
	Performance	2.27	1.27
	Effort	6.00	1.48
	Frustration	3.05	1.80
WP Scale [18]	Attention	7.19	1.44
	Depth Perception Ability	8.12	1.82
	Visual Processing Ability	7.98	1.51
	Haptic Sensing Ability	7.80	1.86
	Audio Processing Ability	4.32	2.56

Note: The questionnaire for measuring cognitive load was constructed as a 5-point Likert scale (1 = “not at all true” to 5 “totally true”). Each row in the table represents the mean and standard deviation of a certain dimension of the cognitive load of all participants in the experimental group

### Correlation analysis

Conrad et al. observed a negative relationship between average EEG alpha and flow experience by conducting a pilot study [21]. However, the limited number of participants in their research might not have a convincing conclusion. Our study tried to reveal the correlation between three kinds of scores.

Firstly, the relation between performance and corresponding cognitive load extracted from EEG is shown in Table 4. In four tasks, the cognitive load had a negative influence on the participants’ performance. For the Pre-FT, the cognitive load score is negatively related to the performance score (*R*^2^= 0.79, *p* = 0.1). For Pre-CRT, the cognitive load score is significantly negatively related to the performance score (*R*^2^= 0.74, *p* < 0.001). The negative relation is also shown in Post-CRT (*R*^2^= 0.61, *p* = 0.05).

**Table 4:** The correlation of performance and cognitive load computed by EEG data

Experiments	Pre-test Performance	Experiments	Post-test Performance
Pre-FT	↓ ^∗^	Post-FT	↓
Pre-CRT	↓ ^∗∗^	Post-CRT	↓^∗∗^

Note: The downward arrow ↓ means negative correlation, **: 0.01 level significant, *: 0.05 level significant

Secondly, the relation between the cognitive scores extracted from scales (CLS) and cognitive load scores computed from EEG (CLE) was also evaluated. The regression R square of CLS and pre-colon resection CLE is 0.68, (*p* < 0.05). The regression R square of CLS and post colon resection CLE is 0.70, (*p* < 0.1). Furthermore, the correlation between performance scores with flow experience showed no significant correlation between self-reported flow experience scores (*p* < 0.05). The correlation between performance scores was also not significantly related to heart rates (*p* < 0.05).

Thirdly, to study the relationship between participants' heart rate and surgical skills during the experiment, the Pearson correlation between surgical skills and heart rate was calculated. The results did not show a significant correlation, as shown in Appendix 1.

## Discussion

The performance scores indicated that VRLS could significantly improve the acquisition of surgical skills. There was no significant difference between the test scores before and after the control group. However, after training on VRLS, the participants could accomplish the same tasks using a shorter time. That means the proficiency of surgical skills has been substantially enhanced. This is similar to the results of a previous study [22], which indicates that virtual surgery simulator can provide learners with a safe and regular practice scene [23]. The combination of simulation and deliberate practice has been proved to be superior to the traditional Halsted approach regarding the acquisition of skills [24]. The traditional method could be replaced with immersive virtual surgical training in the future. At the pre-test phase, the worst-performing dimension was depth perception. After VRLS training, the depth perception becomes the best among the four dimensions. The slightest change dimension was bimanual dexterity.

From the participants’ feedback, the principal source of the cognitive load was the false perception of depth during the operation. Though the depth perception was significantly improved, it is still a significant influence factor. Introducing an immersive training environment might be an alternative scheme. However, that could raise the dizziness problem and cause an uncomfortable training experience.

At present, most studies on the relationship between flow experience and cognitive load focus on online games [25]. Despite the apparent applicability of CLT in a virtual environment, findings from multimedia learning [26] have not been easily replicated within medical simulation training contexts. In some cases, increased cognitive load negatively impacted learning and performance outcomes [27]. This study breaks through this research field by measuring flow experience and cognitive load in medical skill training experiments. The results demonstrate that the participants’ surgery performance relates to their physical-psychological state. Developed skills might indicate lower cognitive load, moderate heart rate, and flow experience. This provides us with additional options to quantitatively measure one’s task skills. We could evaluate one’s performance by monitoring physiological data such as EEG, heart rate. Compared with the traditional subjective method evaluating after experiments [28], this approach could identify the features that increase the participants’ cognitive load in real-time. When integrated with a physiological data detection device, the training course designer could optimize the experiment environment setting and the experiment procedure adjustment. Besides, when medical students suffer from a high cognitive load, the system could help them with guiding information.

Although studies in other areas have shown that flow experiences are associated with performance levels [29], there is no clear relation between flow experience and performance. There is no significant positive correlation between flow experience, heart rate, and surgical skills by analyzing the correlation between flow experience and heart rate. However, there is no apparent correlation between flow experience and surgical skills. Therefore, in our experiment, we could not evaluate the participants' heart flow experience and quantitatively assess the participants' surgical skills by recording physiological data (HR, etc.). When people do not exercise vigorously (such as conducting experiments in our research), heart rate is not a good indicator for evaluating people's flow experience. Heart rate variability (HRV) is a more accurate indicator of HR. Recording a person's heart rate variability may be a better way to evaluate a person's heart flow experience [30].

The improvement of skills proficiency may increase the automation of operation. Then the participants might regard our tasks more efficiently to achieve (frustration = 3.05 in NASA-TLX). This would hinder the acquirement of flow experience. The flow experience might be a state that is not easy to obtain in our study. To get an flow experience, the training system should be optimized with more attractive elements (e.g., guidance information with AR glass).

## Conclusion

Training medical students on VRLS has been considered a promising direction due to its risk-free and high reusability. In this paper, the influence of VRLS on medical students was quantitatively investigated from three aspects: performance evaluation, physiology (heart rate and EEG), and self-reported cognitive load and flow experience. 51 Participants were recruited to conduct pre and post experiments in training boxes. Between the pre and post experiments, the experimental group (41 participants) was trained on VRLS. Their operation video and physiological data (heart rate and electroencephalogram) were recorded. Then their performance was graded by senior surgeons using several scales. Finally, the participants filled questionnaires about their cognitive load and flow experience. The experimental results demonstrate that the VRLS could highly improve medical students’ performance and enable the participants to obtain flow experience with a lower cognitive load. Besides, our study found that the flow experience has no significant relationship with performance and heart rate.

Nevertheless, our work is not without limits. Currently, this study just reveals the correlation between performance and cognitive load. Their exact functional relations such as linearity, non-linearity, or exponential were not investigated yet. Many researchers utilized machine learning to measure and classify cognitive load [31]. This could be a potential research topic in the future.

## Supplementary Information

**Additional file 1.** Appendix 1 is used to show the correlation between pre-test skills and pre-test heart rate and relationship between heart flow experience, heart rate, and surgical skills.

## Data Availability

The data that support the findings of this study are available on request from the corresponding author, [initials]. The data are not publicly available due to [restrictions e.g. their containing information that could compromise the privacy of research participants].

## Abbreviations

AR: Augmented reality
VR: Virtual reality
VRLS: Virtual reality-based laparoscopic surgery simulators
GOALS: Global operative assessment of laparoscopic skills
CL: Cognitive load
CLT: Cognitive load theory
Pre/Post-FL: Pre/post-test of three fundamental surgery skills tasks
Pre/Post-CRT: Pre/post-test of colon resection tasks
EEG: Electroencephalography
CLS: Cognitive scores extracted from scales
CLE: Cognitive load scores computed from EEG
HR: Heart rate
