# Supplementary material for: Quantitative influence and performance analysis of virtual reality laparoscopic surgical training system
Source: BMC Med Educ. 2022 Feb 10;22:92. doi: 10.1186/s12909-022-03150-y (PMC8832780; doi:10.1186/s12909-022-03150-y)
Supplement: Supplementary file 1 — Additional file 1. Appendix 1 is used to show the correlation between pre-test skills and pre-test heart rate and relationship between heart flow experience, heart rate, and surgical skills. [file 12909_2022_3150_MOESM1_ESM.docx]

**Appendix 1**

FT experiments found a negative correlation between pre-test skills and pre-test heart rate (correlation coefficient: -0.24), but not significant (significance: -0.127). There is a negative correlation between the same posterior skills and posterior heart rate (significance: -0.17), but not significant (significance: 0.289). However, in CRT experiments, there was a positive correlation between the participants' surgical skills and heart rate, and it was also not significant (pre-test significance: 0.556, post-test significance: 0.818).

Table 5: The correlation between performance and HR

| Experiments | Pre-test Performance | Experiments | Post-test Performance |
| --- | --- | --- | --- |
| Pre-FT HR | -0.24 (0.127) | Post-FT HR | -0.17 (0.289) |
| Pre-CRT HR | 0.09 (0.556) | Post-CRT HR | 0.04 (0.818) |

To study the relationship between heart flow experience, heart rate, and surgical skills. We first calculated the flow experience score according to the flow experience scale. The flow experience score was used to conduct a Pearson correlation analysis with the average heart rate (Table 6) and surgical skill score (Table 7) in the four pre/post-test experiments. Table 6 shows a positive correlation between flow experience and heart rate. Still, it is not significant (significance>0.05). In Table 7, there is a negative correlation between heart flow experience and heart rate in the basic pre-test and post-large bowel test experiments. There is a positive correlation between flow experience and heart rate in the basic post-test and pre-test experiment, but they are insignificant (significance>0.05).

Table 6: The correlation of flow experience and HR

|  | Pre-FT HR | Pre-CRT HR | Post-FT  HR | Post-CRT HR |
| --- | --- | --- | --- | --- |
| Flow Experience | 0.13(0.42) | 0.02(0.91) | 0.28(0.08) | 0.28(0.07) |

Table 7: The correlation of flow and performance

|  | Pre-FT Performance | Pre-CRT Performance | Post-FT  Performance | Post-CRT Performance |
| --- | --- | --- | --- | --- |
| Flow Experience | -0.04(0.81) | 0.06(0.70) | 0.24(0.13) | -0.14(0.40) |
